# Nutritional and weight status of Indian mother‐child dyads experienced by a natural disaster

**DOI:** 10.1111/mcn.13164

**Published:** 2021-02-25

**Authors:** Natalia Nowak‐Szczepanska, Aleksandra Gomula, Raja Chakraborty, Slawomir Koziel

**Affiliations:** ^1^ Department of Anthropology, Hirszfeld Institute of Immunology and Experimental Therapy Polish Academy of Sciences Wroclaw Poland; ^2^ Department of Anthropology Dinabandhu Mahavidyalaya Bongaon West Bengal India

**Keywords:** arm circumference, body mass index, children, natural disaster, nutritional status, prenatal programming

## Abstract

Natural disasters have detrimental effects not only on local infrastructure in an affected population but may also have an impact on the human biological condition, particularly during critical periods of life. This study aimed to assess the nutritional and weight status of women and their children who had experienced cyclone Aila prenatally and postnatally in comparison with a non‐affected neighbouring group. The study sample involved *N* = 597 dyads consisting of mothers and their prepubertal children prenatally or postnatally (during infancy) exposed to a natural disaster and a control group from a neighbouring region (West Bengal, India). The analysed anthropometric indices involved body mass index (BMI) and mid‐upper arm circumference (MUAC). Moreover, several socioeconomic characteristics were collected (mother's and father's education, family size and family income). Analyses revealed that the group factor (Aila‐exposed or non‐exposed groups) had the highest impact on both children's and their mothers' BMI and MUAC (*p* < 0.001) in comparison with socioeconomic variables. Surprisingly, both mothers and their children revealed deteriorated nutritional and relative weight status several years after the occurrence of cyclone Aila, which is in opposition to the results obtained in developed countries, where prenatal maternal stress caused by the natural disaster led to the subsequent higher risk of excessive weight in affected children.

**Key messages**

- Mothers and prepubertal children experienced by cyclone Aila (during pregnancy and prenatally or postnatally, respectively) reveal subsequent deteriorated nutritional and relative weight status compared to their counterparts living in a similar, neighbouring area, but non‐exposed to the natural disaster. This pattern is different in comparison with developed societies. Thus, low‐income, developing populations should implement adequate emergency policies after the occurrence of any natural disaster in energy‐restricted environments.
- The experience of a natural disaster seems to have a greater effect on nutritional and relative weight status compared to the current socioeconomic conditions in Indian women and their children.
- There was no difference in BMI between children prenatally or postnatally exposed to a natural disaster, whereas MUAC had lower values in children exposed to Aila *in utero*, compared to those experienced during infancy (however, MUAC in postnatally experienced children was still significantly lower than in non‐exposed counterparts).

## 1 INTRODUCTION

The developmental programming hypothesis (e.g., Barker, 1995; Godfrey & Barker, 2001) indicates the importance of maternal conditions for foetal, as well as later growth and development. Disproportionate foetal growth caused particularly by nutritional deficiencies or environmental stress could be associated with metabolic or endocrine dysregulation resulting in negative health outcomes in adulthood, such as coronary heart disease or metabolic disorders (Barker, 1995; Gluckman et al., 2005). Animal studies show how severe prenatal maternal stress experience (Beydoun & Saftlas, 2008) is related to a higher level of maternal glucocorticoids which pass to the foetus through the placenta and affect foetal development, resulting in different biological outcomes.

In humans, it is impossible to conduct laboratory experiments to assess the effects of severe prenatal maternal stress on foetal growth. However, human experiences of certain spontaneous and natural phenomena might provide such opportunity, for example, stressful life events, such as divorce or death of a close family member, war affliction or natural disasters (King & Laplante, 2015). Particularly, pregnant women may experience remarkable stress during a natural disaster affecting both her and her offspring. Some researchers focused mostly on the subsequent effects observed in offspring and noted that severe maternal stress caused by a natural disaster may result in, for instance, worse cognitive development, weaker language skills, higher dermatoglyphic asymmetry or even altered brain structure in children (King & Laplante, 2015). Similarly, growth and nutritional status of children, who were exposed to prenatal maternal stress due to a natural disaster, may be affected and can result in a higher risk of obesity, subsequently in childhood and adolescence (Cao‐Lei et al., 2015; Dancause et al., 2012, 2015).

Body mass index (BMI = weight [kg]/height [m]^2^) is a commonly used indicator of relative weight and is widely applicable for inter‐population comparisons of levels of overweight, obesity and underweight (e.g., NCD‐RisC, 2017; Ng et al., 2014). However, it is not an ideal measure as it includes different types of tissues, that is, muscle and adipose, as well as all the components of the total body mass, such as different organs and bones (see also Peterson et al., 2017). It should be also noted that weight is not directly proportional to height squared in children and adolescents, due to their variable tempo and patterns of growth; thus, BMI has to be standardized on children's age and sex (e.g., Peterson et al., 2017). Alternatively, a mid‐upper arm circumference (MUAC) may be a more appropriate indicator of nutritional status, because it is highly associated with the content of adipose tissue, particularly in children (Chomtho et al., 2006). MUAC also reliably reflects malnutrition, mainly among children from low‐income populations (e.g., Briend et al., 2016; Debnath et al., 2017; Isanaka et al., 2019; Taneja et al., 2018).

Both BMI and MUAC in children may be affected by several environmental and biological factors, such as genetic makeup, maternal weight status, nutritional resources or socioeconomic conditions. Among environmental factors, socioeconomic aspects might play a substantial and significant role in respect of weight status in both children and adults. Numerous studies have found significant differences in relative weight between the categories of the most common socioeconomic variables: urban *versus* rural locations (e.g., NCD‐RisC, 2019), higher *versus* lower‐educated families (e.g., Lamerz et al., 2005), higher *versus* lower income (e.g., Bammann et al., 2013), considering social inequalities (e.g., Wells et al., 2012) and smaller *versus* larger families (e.g., Chen & Escarce, 2010). However, the magnitude and direction of these socioeconomic differences often depend on whether they are observed in developed or developing populations around the world (Dinsa et al., 2012; Monteiro et al., 2004; Pampel et al., 2012; Silventoinen et al., 2019). For instance, higher social classes have higher relative weight in developing, particularly low‐income societies, where weight status is positively related to individual income. In contrast, in developed countries, higher socioeconomic groups tend to be more focused on healthy lifestyles and are at lower risk of having excess body weight (e.g., Dinsa et al., 2012; Monteiro et al., 2004).

In developed countries, it has been already shown that prenatal exposure to natural disasters, such as the Quebec Ice Storm in 1998, the Iowa floods in 2008 or the Queensland floods in 2011, was associated with significant changes in growth, development and weight status in offspring (Dancause et al., 2012, 2015; King et al., 2015). Because developed and developing societies may have different opportunities and resource set up to deal with the effects of a natural disaster, different biological outcomes may be expected in high‐income *versus* low‐income populations exposed to a natural disaster. Noteworthy differences should arise particularly during the critical periods of development, that is, during prenatal and infant growth. However, some significant and long‐term biological effects may appear also in mothers, who were pregnant and likely to have been extremely stressed when exposed to a natural disaster. Still, up to date, this issue has not yet been investigated.

Therefore, this study aimed to compare the nutritional and weight status of women and their prepubertal children from different groups in respect of the severe tropical cyclone, Aila, that struck the eastern parts of India, particularly the Sundarbans area in West Bengal, on May 25, 2009. These study groups of children and their mothers included (i) those exposed to a natural disaster during pregnancy (prenatally), (ii) those exposed to the natural disaster during infancy and (iii) those not exposed to cyclone Aila. Our research hypothesis argued that both women and their children experiencing the severe cyclone, particularly during pregnancy and prenatal stage, respectively, would have significantly different nutritional and weight status several years after the natural disaster compared to their counterparts from a neighbouring region that did not experience cyclone Aila. Concurrently, we hypothesize that a pattern of the differences in nutritional and weight status in women and their children exposed to a natural disaster in a developing society may differ from those observed in the high‐income, developed populations.

## 2 METHODS

### 2.1 Study areas

This cross‐sectional study was conducted in two areas in the state of West Bengal in the eastern part of India (for detailed maps of the study areas, see, e.g., Mukhopadhyay, 2009; for a track of the Aila cyclone, see also Mitra et al., 2011). The experimental population lived in the Sundarbans delta region where the effect of the tropical cyclone, Aila, was the most severe. This natural disaster persisted in Sundarbans region from May 23 to May 26, 2009, whereas the storm reached its peak on the May 25 with a speed of 110 km/h. It was classified as a severe cyclonic storm, which had caused massive destruction of the surrounding environment, including coastal erosion and the intrusion of seawater (for more details, see Mitra et al., 2011). Inhabitants of the affected region lost their properties, infrastructure and experienced extreme physical adversity (e.g., Mondal et al., 2016). Our study participants lived on two islands, namely, Satjelia and Kumirmari, under the community development block (CDB), called Gosaba (in the district of South 24 Parganas). These were the most affected islands, in terms of severity of the damage due to the cyclone. Data were collected from 22 schools (out of total 30) in Satjelia Island and all the 13 schools in Kumirmari Island.

The control group was recruited from the rural areas of Bongaon CDB under the Bongaon sub‐division of the neighbouring district of North 24 Parganas. These areas were broadly adjacent to the experimental areas. Three administrative rural units, called the Gram Panchayats (GP), and 21 primary schools (out of total 34) within these three GPs, were selected by simple random procedures. In this region, Aila did not have a significant impact, except for a mild storm that was normal for the season, and no flood situation had occurred.

### 2.2 The participants

This study was conducted on three different groups of mothers and their children:

- Aila‐exposed women and their offspring (*N* = 238), who were pregnant and intrauterine, respectively, on the day of cyclone. These children were born between June 2009 and February 2010.
- Aila‐exposed women and their children (*N* = 138), who were newly mothers and infants, respectively, on the day of cyclone. These children were born during the few months preceding Aila and faced all the post‐disaster hazards during their early postnatal growth. These mother‐children dyads lived in the same areas as did the first group.
- The control group: not exposed to Aila (*N* = 221) and belonging to the same birth cohort, that is, was intrauterine on the day of cyclone and born between June 2009 and February 2010. They were recruited from the villages of the neighbouring district that did not face the cyclone. The people of these areas were very similar with the Sundarbans people in respect of the origin, culture, language and lifestyle.

During this survey, the children were at their pre‐pubertal period (7–10 years of age; mean = 8.43 years of age, SD = 0.52), whereas mothers' age varied from 23 to 47 years of age (mean = 29.94 years of age, SD = 3.81).

### 2.3 The sampling procedure

The study followed a multistage sampling strategy. First, two islands were selected on the basis of the published information on the severity of the cyclone and damage of life and properties. Several pilot visits by one of the principal investigators of the project confirmed this information before final selection. All children studying in the primary schools on those islands and qualifying for the recruitment criteria, as described above, as well as their mothers were the target participants. Analogously, children and their mothers from a control group in a neighbouring area were examined. Each school was requested to participate in this study, and a visit was fixed by mutual agreement as the presence of children along with their mothers fulfilling the criteria was ensured. On the day of the visit, the field investigators verified dates of birth by appropriate official documents and finally included the mothers and children for the study. Children having any kind of diseases or physical deformity that could influence any measurement were not included. Informed consent from the mother of each child was obtained before examination.

### 2.4 Measurements and socioeconomic data

Height, body weight and MUAC of mothers and their children were measured by a trained field investigator following the standard measuring protocol (Martin & Saller, 1957). Height and mid‐upper arm circumference were measured by anthropometer and anthropometric tape, respectively, whereas weight was assessed using a weight scale. A relative weight status was calculated by BMI (kg/m^2^) and nutritional status was evaluated by MUAC (cm). For descriptive purposes, BMI and MUAC in children were standardized for age according to LMS parameters (*L* for the skewness, *M* for median value and *S* for the generalized coefficient of variation), separately for both sexes (Cole et al., 2000; Mramba et al., 2017, respectively, for BMI and MUAC). Descriptive statistics of BMI and MUAC in mothers and their age‐standardized values in children are presented in Table 1. In analyses of children's BMI and MUAC, the mothers' weight status as the independent variable was categorized as non‐overweight (BMI < 25.00 kg/m^2^) or overweight (BMI ≥ 25.00 kg/m^2^). Three groups of examined children did not differ significantly in their birth weight; thus, this variable was not included in further analyses. Socioeconomic data were collected using a pretested questionnaire by interviewing the mothers. Basic socio‐demographic information, as well as weight status of mothers and their children (in children, weight status was based on IOTF cut‐offs; Cole et al., 2000; Cole et al., 2007) are shown in Table 2. All participants were inhabitants of rural settlements. Socioeconomic information included years of education and type of school attended, the total number of family members in a household and total family income per month. Mother's and father's education was categorized as not educated, at most primary and at most secondary. As the families were very often composed not only of parents and their children but also other relatives living together in one household, forming relatively large families, this variable was divided into three categories (based on the tertiles of its distribution): up to 3 family members, between four and five family members and more than five family members. The family income per month was divided by the number of family members and classified as below or above the mean value of monthly family income *per capita* (in our sample, mean monthly income *per capita* was calculated as 1297.95 INR).

**TABLE 1:** Descriptive statistics of BMI (kg/m^2^) and MUAC (cm) in mothers and standardized for age values of BMI (Z‐BMI) and MUAC (Z‐MUAC) in children from different groups: Aila‐exposed prenatally, Aila‐exposed postnatally and control group

	*N*	*M* (*SD*)	*Me* (Q1;Q3)	*N*	*M* (SD)	*Me* (Q1;Q3)
Group	Mothers' BMI			Mothers' MUAC		
Aila prenatally	238	23.00 (3.64)	22.84 (20.43;25.56)	238	24.59 (2.70)	24.50 (22.90; 26.40)
Aila postnatally	138	22.52 (3.14)	21.79 (19.79;24.53)	138	24.43 (2.97)	24.30 (22.30; 26.60)
Control	221	25.44 (4.15)	25.03 (22.37;28.23)	221	25.96 (3.06)	26.10 (23.80; 28.20)
Group	Children's Z‐BMI			Children's Z‐MUAC		
Aila prenatally	238	−1.27 (1.48)	−1.10 (−1.99;−0.29)	237	−1.92 (1.13)	−1.88 (−2.55;−1.19)
Aila postnatally	138	−1.45 (1.15)	−1.42 (−2.20;−0.73)	138	−1.90 (1.17)	−1.87 (−2.45;−1.27)
Control	221	−0.12 (1.23)	0.05 (−0.99;0.79)	221	−1.15 (1.29)	−1.27 (−2.06;−0.39)

Abbreviations: M, mean; Me, median; N, number; Q1;Q3, lower and upper quartiles; SD = standard deviation.

**TABLE 2:** Sociodemographic data and weight status of the study sample (mothers and their children Aila‐exposed prenatally, Aila‐exposed postnatally and a non‐exposed control group)

	Aila prenatally	Aila postnatally	Control
Mothers	*M* ± *SD*/*N* (%)	*M* ± *SD*/*N* (%)	*M* ± *SD*/*N* (%)
Age (years)	29.21 ± 3.73	31.00 ± 3.48	30.06 ± 3.94
*Mother's education*			
Not educated	9 (3.78%)	27 (19.57%)	25 (11.31%)
At most primary	163 (68.49%)	93 (67.39%)	122 (55.20%)
At most secondary	66 (27.73%)	18 (13.04%)	74 (33.48%)
*Family income*			
Below mean value	163 (68.49%)	117 (84.78%)	106 (47.96%)
Above mean value	75 (31.51%)	21 (15.22%)	115 (52.04%)
*Family size*			
Up to 3 members	30 (12.60%)	8 (5.80%)	30 (13.58%)
4–5 family members	165 (69.33%)	99 (71.74%)	142 (64.25%)
> 5 family members	43 (18.07%)	31 (22.46%)	49 (22.17%)
*Weight status*			
Underweight	26 (10.92%)	5 (3.62%)	4 (1.81%)
Normal weight	145 (60.92%)	102 (73.91%)	102 (46.15%)
Overweight	59 (24.79%)	29 (21.01%)	84 (38.01%)
Obese	8 (3.36%)	2 (1.45%)	31 (14.03%)

Children	*M* ± *SD*/*N* (%)	*M* ± *SD*/*N* (%)	*M* ± *SD*/*N* (%)
Age (years)	8.05 ± 0.23	9.22 ± 0.30	8.34 ± 0.24
*Sex*			
Boys	123 (51.68%)	70 (50.72%)	109 (49.32%)
Girls	115 (48.32%)	68 (49.28%)	112 (50.68%)
*Father's education*			
Not educated	26 (10.92%)	18 (13.04%)	35 (15.84%)
At most primary	124 (52.10%)	75 (54.35%)	107 (48.42%)
At most secondary	88 (36.98%)	45 (32.61%)	79 (35.74%)
*Weight status*			
Underweight	102 (42.86%)	78 (56.52%)	43 (19.46%)
Normal weight	131 (55.04%)	58 (42.03%)	143 (64.71%)
Overweight	4 (1.68%)	2 (1.45%)	26 (11.76%)
Obese	1 (0.42%)	0	9 (4.07%)

Abbreviations: M, mean; *N*, number; SD, standard deviation.

### 2.5 Statistical analyses

To assess the effects of Aila‐experience (group factor) and other independent variables (categorized: mother's and father's education, family size, family income per capita and, in children's analyses, sex and mother's weight status) on BMI and MUAC (dependent variables) in mothers and their children, the multi‐way Analysis of Covariance (MANCOVA) was conducted controlling for age of participants as a covariate. Due to the skewed distribution (except for women's MUAC with a normal distribution), most of the analysed variables had to be transformed (using Box‐Cox transformation technique). Tukey's post hoc tests for unequal sample sizes were performed to assess the significance of the differences in analysed measures between the categories of independent factors. The effect size was evaluated by partial eta squared parameters (η^2^). The required significance level was assumed at *p* < 0.05. Analyses were conducted using Statistica 13.1 software.

### 2.6 Ethical considerations

Ethical approval was obtained from the Institutional Ethics Committee for Research on Human Subjects, West Bengal State University, West Bengal, India (approval no. WBSU/IEC/14/03, dated 13.11.2017). Moreover, the Protection of Children from Sexual Offences (POCSO) Act of India was also adhered to. All procedures were performed according to the Declaration of Helsinki. All participants gave their informed consent prior to the examinations.

## 3 RESULTS

**FIGURE 1:**
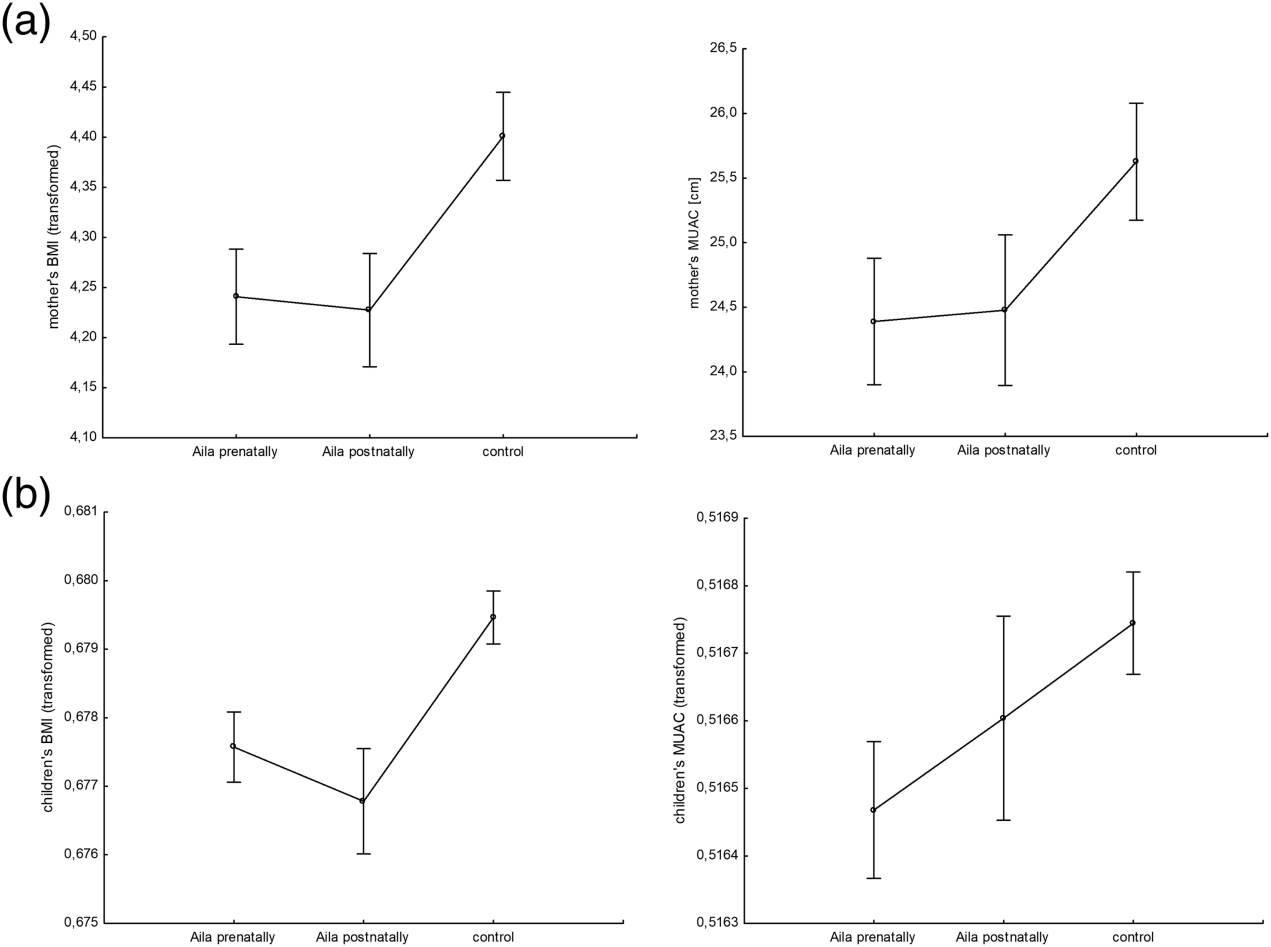
Body mass index = BMI (kg/m^2^; Box‐Cox transformed values) and mid‐upper arm circumference = MUAC (cm; raw values or Box‐Cox transformed values) in (a) mothers and (b) their children exposed to cyclone Aila prenatally, postnatally and from a non‐exposed control group

For mothers' BMI, significant effects of the group factor (*F*_(2,586)_ = 22.51, *p* < 0.001), fathers' education (*F*_(2,586)_ = 4.45, *p* < 0.05) and family income (*F*_(1,586)_ = 12.34, *p* < 0.001) were observed after controlling for mothers' age (*p* < 0.05). This model explained *R* ^2^ = 14.80% of the variance of the analysed variable. Tukey's post hoc tests showed that the mothers of both prenatally and postnatally Aila‐exposed children had significantly lower BMI than the mothers of the control group (both *p* < 0.001; Figure 1a), whereas the mothers of prenatally and postnatally Aila‐exposed children did not differ significantly in BMI (*p* > 0.05). Moreover, mothers with at most secondary level of education had significantly higher BMI than mothers with at most primary level of education and those mothers not educated at all (both *p* < 0.01). Mothers having family income *per capita* above mean value had higher BMI compared to mothers from below mean value category of family income (*p* < 0.001). It should be noted that in the MANCOVA model, with the mothers' BMI as the outcome, the effect size was the highest for the group factor (partial η^2^ = 0.071; Table 3).

**TABLE 3:** Results of multi‐way analysis of covariance for BMI and MUAC in mothers

Effect	Mothers' BMI			Mothers' MUAC		
	*F*	*p* value	Partial η^2^	*F*	*p* value	Partial η^2^
Age	5.75	0.017^*^	0.010	6.12	0.014^*^	0.010
Group factor	22.51	<0.001^***^	0.071	11.35	<0.001^***^	0.037
Father's education	4.45	0.012^*^	0.015	1.60	0.204	0.005
Mother's education	1.44	0.239	0.005	4.30	0.014^*^	0.014
Family size	0.57	0.564	0.002	0.09	0.911	<0.001
Family income	12.34	0.001^**^	0.021	8.22	0.004^**^	0.014
	*R* ^2^ = 16.23%; adj. *R* ^2^ = 14.80%			*R* ^2^ = 10.80%; adj. *R* ^2^ = 9.28%		

*** *p* < 0.001.

** *p* < 0.01.

* *p* < 0.05.

Regarding mothers' MUAC, significant effects of the group factor (*F*_(2,586)_ = 11.35, *p* < 0.001), mothers' education (*F*_(2,586)_ = 4.30, *p* < 0.05) and family income (*F*_(1,586)_ = 8.22, *p* < 0.01) were noted after controlling for mothers' age (*p* < 0.05). The above‐mentioned model explained *R* ^2^ = 9.28% of the variance of mothers' MUAC. Post hoc comparisons revealed that mothers of prenatally and postnatally Aila‐exposed children had significantly lower MUAC than mothers from the control group (*p* < 0.001; Figure 1a). However, both these Aila‐exposed groups did not differ significantly in MUAC. With respect to education, mothers who had received at most secondary education had significantly higher MUAC than the non‐educated group of mothers (*p* < 0.01). Mothers with family income *per capita* above mean value had significantly higher MUAC than those with family income below mean value (*p* < 0.001). Similarly, as in case of BMI, the highest effect size for MUAC was observed for the group factor (partial η^2^ = 0.037; Table 3).

In relation to children's BMI, significant effects of the group factor (*F*_(2,584)_ = 48.16, *p* < 0.001), mothers' overweight status (*F*_(1,584)_ = 16.33, *p* < 0.001) and fathers' education (*F*_(2,584)_ = 3.76, *p* < 0.05) were observed after controlling for children's age (*p* < 0.05). This model explained *R* ^2^ = 20.34% of the variance of children's BMI. Tukey's post hoc tests revealed that the lowest BMI had the prenatally and postnatally Aila‐exposed children compared to the control group (*p* < 0.001; Figure 1b), whereas both Aila‐exposed groups did not differ significantly in BMI (*p* > 0.05). Children of overweight mothers had higher BMI than their counterparts of non‐overweight mothers (*p* < 0.001). The highest BMI was observed among children whose fathers had received at most secondary level of education. However, this difference was statistically significant while comparing children of fathers who had at most primary education level (*p* < 0.01). In the MANCOVA model for children's BMI, the highest effect size was noted for the group factor (partial η^2^ = 0.142; Table 4).

**TABLE 4:** Results of multi‐way analysis of covariance for BMI and MUAC in children

Effect	Children's BMI			Children's MUAC		
	*F*	*p* value	Partial η^2^	*F*	*p* value	Partial η^2^
Age	4.14	0.042^*^	0.007	2.22	0.137	0.004
Sex	0.25	0.615	<0.001	0.02	0.878	<0.001
Group	48.16	<0.001^***^	0.142	16.39	<0.001^***^	0.053
Mother's overweight status	16.33	<0.001^***^	0.027	23.59	<0.001^***^	0.039
Father's education	3.76	0.024^*^	0.013	0.12	0.884	<0.001
Mother's education	0.70	0.495	0.002	5.36	0.005^**^	0.018
Family size	0.17	0.844	0.001	0.16	0.853	<0.001
Family income	0.25	0.617	<0.001	2.15	0.143	0.004
	*R* ^2^ = 21.94%; adj. *R* ^2^ = 20.34%			*R* ^2^ = 16.99%; adj. *R* ^2^ = 15.28%		

*** *p* < 0.001.

** *p* < 0.01.

* *p* < 0.05.

For children's MUAC, significant effects were found for the group factor (*F*_(2,583)_ = 16.39, *p* < 0.001), mothers' overweight status (*F*_(1,583)_ = 23.59, *p* < 0.001) and mothers' education (*F*_(2,583)_ = 5.36, *p* < 0.01) after controlling for children's age (which was non‐significant, *p* = 0.14). This model explained *R* ^2^ = 15.28% of the variance of children's MUAC. Post hoc comparisons revealed that the prenatally Aila‐exposed children showed the lowest MUAC (Figure 1b) compared to both postnatally Aila‐exposed group (*p* < 0.001), as well as the control group (*p* < 0.001). Nevertheless, MUAC was still significantly lower also in the postnatally Aila‐exposed group in comparison with the control group (*p* < 0.01). Children of overweight mothers had significantly higher MUAC than children of mothers with a non‐overweight status (*p* < 0.001). The value of MUAC in children whose mothers had at most secondary education level was significantly higher than that of the children of mothers with at most primary level of education and non‐educated mothers (both *p* < 0.05). In the corresponding MANCOVA model for children's MUAC, the effect size was the highest for the group factor (partial η^2^ = 0.053; Table 4).

## 4 DISCUSSION

This study revealed significantly lower values of both BMI and MUAC among mothers and their prepubertal children who were exposed either *in utero* or during infancy to the severe cyclone Aila in comparison with the control group which had not experienced the disaster. The results confirmed the research hypothesis showing that both mothers' and children's nutritional and weight status were significantly different, even after some years of experiencing a natural disaster, compared to the nutritional and weight status of inhabitants from a non‐exposed group to Aila, residing in a neighbouring region in India. However, an important issue of the present study was that the deteriorated nutritional and weight status in children exposed to the severe cyclone and suffering its after effects *in utero* or during their infancy was in contrast to the results of other previous studies analysing the effects of natural disaster during prenatal development. In those studies, children tended to have higher relative weight afterwards and were at greater risk of obesity in subsequent years (Cao‐Lei et al., 2015; Dancause et al., 2012, 2015; Kroska et al., 2018). Yet it should be noted that the above‐mentioned surveys had been conducted in high‐income, developed countries where the potential for nutritional recovery after a natural disaster could be intuitively much more conducive, resulting even in excess weight, probably due to the changes of metabolic regulations in these affected children.

Many studies revealed that the adverse prenatal conditions, caused by the prenatal maternal stress, may modify foetal metabolic pathways (Cao‐Lei et al., 2015; Gluckman et al., 2005; Gluckman & Hanson, 2004), for instance, in the direction of subsequent insulin resistance and visceral fat accumulation in later years of development. These modifications may be associated with excess body fat in an energy‐dense environment. Generally, developed countries have a higher level of living conditions, better access to food resources and more advanced infrastructure and healthcare. Thus, these favourable circumstances may enhance the fulfilment of the developmental programming directed to the accumulation of relatively greater amount of adipose tissue from the energy‐rich environment. In contrast, a population from a relatively energy‐restricted environment, such as poor rural areas severely affected by a natural disaster in a developing country like India, probably did not have sufficient resources to start a nutritional recovery after the catastrophic event. Therefore, it can be speculated that a prenatal or early postnatal programming, developed during an experience of a natural disaster, lost its significance. This deductive hypothesis, based on our findings, to some extent, corresponds to the results of the Canadian Project Ice Storm study, where a subsequent higher risk of obesity in children, exposed prenatally to a natural disaster, was observed mostly in higher socioeconomic groups, not in the lower ones (Dancause et al., 2012).

Due to the higher effect size of the group factor affecting BMI and MUAC in children compared to those observed in mothers, the offspring seems to be more severely affected by a natural disaster than mothers. Thus, the biological effects of a natural disaster were seemed to be more noticeable when it occurred during the critical periods of development, such as prenatal growth or infancy (in our study with similar effects on both, because there were no significant differences in the anthropometric indicators between groups prenatally and postnatally exposed to Aila, except for MUAC in children). The prenatal and early‐life conditions (such as the undernutrition leading to lower birth weight or lower infant weight) were revealed by many other researchers as of particular importance for later human biological status and health (such as obesity, coronary heart diseases or metabolic diseases), even in the course of the later adulthood (e.g., Barker, 2007; Eriksson, 2016; Gluckman et al., 2005; Hoffman et al., 2017). However, in our study, stressful life events (due to the severe cyclone) during the critical periods of life (*in utero* and infancy) resulted in a deteriorated nutritional and weight status in prepubertal children of a low‐income population in India. Further studies are needed to verify whether potentially increased adiposity would appear in later periods of life in these children, for instance, during adolescence.

Another interesting aspect of this study was related to the differences in the effect sizes between particular independent factors, that is, in the influence of these factors on the nutritional or weight status in children and their mothers. The tropical cyclone Aila had particularly detrimental effects on human beings in the studied region of India, both ecologically, physically and psychologically, due to the magnitude of this traumatic event and because of limited access to healthcare and a lack of appropriate infrastructure in this location (see also Bhattacharyya et al., 2010; Mitra et al., 2011). Environmental factors during a natural disaster seem to be more important in determining current biological status than the socioeconomic variables or even maternal weight status. Researchers have noted that mother's BMI is one of the most significant determinants of children's weight, as well as other outcomes in offspring, both when maternal BMI values are too low or too high (e.g., Hernández‐Valero et al., 2007; Kaar et al., 2014; Kalk et al., 2009). Our results, although including only the current BMI status of the mothers, corresponded to this association, because mother's weight status was significantly correlated with both BMI and MUAC in children.

Of further importance, the socioeconomic factors remained significant in respect of children's or mothers' BMI and MUAC, in the direction that was observed also among other low‐income, developing countries (e.g., Dinsa et al., 2012; Monteiro et al., 2004), where higher educated, smaller families with a higher household income had higher values of relative weight (e.g., Monteiro et al., 2004; Pampel et al., 2012). Nevertheless, relatively lower strength of observed associations for BMI and MUAC with the current socioeconomic conditions, compared to the past effect of a natural disaster, was somewhat surprising, because some of the socioeconomic factors were found in many other studies to significantly affect relative weight or nutritional status (e.g., Bouthoorn et al., 2014; Lamerz et al., 2005; Nowak‐Szczepanska et al., 2020; Pampel et al., 2012; Shrewsbury & Wardle, 2008). Particularly, parental education has been implicated to be one of the most significant determinants of children's weight status, especially where mother's higher education leads to improvement in the family's dietary habits, whereas father's higher education provides better access to food resources (see also Frost et al., 2005; Lamerz et al., 2005; Moestue & Huttly, 2008).

It should be noted that, in general, undernutrition is one of the most serious health concerns in India (compare Ramesh et al., 2017; Khanra et al., 2020). Therefore, adequate policies should be implemented to improve socioeconomic conditions in Indian families, especially among those affected by natural disasters. Particularly, the studied regions of South 24 Parganas Districts present a significantly high prevalence of undernutrition among preschool children (Biswas et al., 2018; Giri et al., 2017). The parental socioeconomic conditions in India remain a significant factor affecting children's growth and development. Furthermore, in the Indian state under study (West Bengal), previous research confirmed the important role of maternal education in shaping children's nutritional status, where a mother's lower education level was strongly associated with an increased risk of undernutrition in children (e.g., Khanra et al., 2020). In our study, a higher level of mother's education was also positively associated with mothers' and children's MUAC, whereas father's higher level of education positively affected mothers' and children's BMI. Along with policies promoting higher socioeconomic status in poorer families, a much higher standard of drinking water, sanitation and hygiene practices (WASH practice) should be provided and implemented more seriously in rural India, because these factors may also affect nutritional status in children and adolescents (compare, e.g., Chattopadhyay et al., 2019; Khanra et al., 2020).

The lack of difference in values of BMI between children, prenatally *versus* postnatally exposed to cyclone Aila, allows for the proposition that the effect of environmental stress during prenatal growth, and infancy is closely similar in both these developmental periods for subsequent relative weight status in later childhood. Therefore, it seems that both effects of prenatal and early postnatal developmental plasticity are interdependent for the constitution of this biological indicator (see also Gluckman et al., 2005). On the other hand, a significant difference was observed in terms of lower values of MUAC in children prenatally exposed to cyclone Aila compared to those exposed postnatally (however, the last group had still lower MUAC than the non‐exposed control group). It is possible that MUAC, compared to BMI, may be more sensitive during the prenatal period to the detrimental effects of environmental stress on later nutritional status (compare also Nowak‐Szczepanska et al., 2019), while, if stress was experienced during infancy, this anthropometric trait would be, at least partially, recovered.

One of the limitations of this study involved the assessment of the results of a natural disaster related only to the effects of the objective stressful event, whereas no information was obtained with respect to the subjective level of distress caused by this catastrophic event among women and their children. Some researchers have observed that objective hardship and subjective distress may have different effects on children's biological outcomes in response to natural disasters (e.g., Dancause et al., 2011). Thus, further studies should include both aspects of maternal stress. Another limitation is related to the studied period of the development of investigated children, who were at their pre‐pubertal age. It was a time between 8 and 10 years after the occurrence of cyclone Aila. Therefore, we have not been able to identify the nutritional and weight status of these children during adolescence. It is possible that during this subsequent developmental period, Aila‐exposed boys and girls would be able to catch‐up their relative weight and diminish the differences in nutritional status compared to their non‐exposed peers. This issue might be important for potential future follow‐up investigations. On the other hand, mothers experiencing Aila, during pregnancy or as newly mothers, showed their lower values of relative weight and nutritional status indicators, in comparison with non‐exposed mothers. Therefore, it seems that a natural disaster might have a long‐lasting detrimental effect on the biological condition, albeit with different strengths, during different periods of human life. Another limitation was associated with the lack of some information: about dietary habits, other nutritional indicators and about food security issues and water, sanitation and hygiene practices (WASH practices), which were not obtained during and after Aila. Nevertheless, in this study, we analysed nutritional and relative weight status as the surrogate indicators of nutritional conditions, and in each model, the experience of the natural disaster was a stronger predictor of BMI or MUAC values than the others, such as socioeconomic factors. Finally, our data missed a longitudinal character that could provide information concerning long‐term changes in nutritional conditions.

## 5 CONCLUSIONS

The biological effects of a natural disaster are particularly pronounced after exposure to this event during the critical periods of human development, that is, prenatal period and infancy. Children from a low‐income, developing country revealed a somewhat different pattern of nutritional and weight status compared to their peers exposed prenatally to a natural disaster in developed countries. The latter were, subsequently, at higher risk of excess weight. Moreover, our Indian study revealed that not only children but also mothers who were pregnant during a natural disaster presented lower values of BMI and MUAC many years after the exposure compared to the non‐affected control group. A deteriorated nutritional and weight status of children and their mothers from a developing population who experienced a natural disaster may indicate a particular need for emergency interventions, as well as adequate social policies that should be implemented in affected low‐income countries.

## CONFLICTS OF INTEREST

The authors declare that they have no conflicts of interest.

## CONTRIBUTIONS

NNS edited the database, analysed and interpreted the data and drafted the manuscript. AG edited the database, provided critical comments and edited the manuscript for intellectual content. RC supervised the data collection, prepared database and edited and revised the manuscript. SK organized, arranged and coordinated the study; edited the database and provided critical comments. All authors have read, revised and approved the final manuscript.

## Data Availability

The data that support the findings of this study are available on reasonable request from the corresponding author. The data are not publicly available due to privacy or ethical restrictions.
